# Long-term efficacy of Boswellia serrata in 4 patients with chronic cluster headache

**DOI:** 10.1186/1129-2377-14-S1-P37

**Published:** 2013-02-21

**Authors:** C Lampl, B Haider, C Schweiger

**Affiliations:** 1 Hospital of Sisters of Mercy, Linz, Austria; 2 LNK Wagner Jauregg, Linz, Austria; 3 Barmherzige Brüder Linz, Austria

## Background

Cluster headache (CH) is an extremely severe and debilitating trigemino-autonomic pain syndrome.About 10% of patients with CH manifest a chronic form CH (CCH). Extracts of Boswellia serrata have been clinically studied for the treatment of many inflammatory conditions such as osteoarthritis and rheumatoid arthritis (3). The resin from Boswellia Serrata contains a number of biological actives called pentacyclic triterpene acids, which give the extract its anti-inflammatory and analgesic properties, with boswellic acid the major active ingredient (4). These acids have been demonstrated to interfere with the body’s natural inflammatory response by inhibiting cytokines and leukocyte activity. The present study aims to evaluate the long-term efficacy of Boswellia Serrata (Sallaki H15) on headaches and disturbed sleep in patients with CCH.

## Results

In an open-label study, 4 patients with CCH and disturbed sleep received oral Boswellia Serrata.

## Conclusion

The effects were long-lasting in 3 patients (mean 15 months) and transient (6 months) in one patient. The rapid improvement of nocturnal pain within weeks is similar to the analgetic effect observed in recent trials using Boswellia Serrata in cancer pain The mechanisms of how Boswellia Serrata reduces pain in CCH remain unclear. Boswellic acids, constituents of Boswellia extract have subsequently been identified as selective redox independent noncompetitive inhibitors of both 5-lipoxygenase, the key enzyme in leukotriene biosynthesis and human leukocyte elastase. Proinflammatory cytokines, such as leukotrienes, are known to play a role in the pathophysiology of CH. This study provides Class IV evidence that oral Boswellia Serrata (Sallaki H15) reduces the intensity and frequency of headaches in patients with CCH.

## Competing interests

None
